# The structure of social support: a multilevel analysis of the personal networks of people with severe mental disorders

**DOI:** 10.1186/s12888-022-04278-3

**Published:** 2022-11-14

**Authors:** François Wyngaerden, Raffaele Vacca, Vincent Dubois, Vincent Lorant

**Affiliations:** 1 Institute of Health and Society, Université catholique de Louvain, Clos Chapelle-aux-Champs, B1.30.15, B-1200 Brussels, Belgium; 2 Epsylon, Network of Psychiatric Services, Avenue Jacques Pastur 49, B-1180 Brussels, Belgium; 3 Department of Sociology and Criminology & Law, University of Florida, 3219 Turlington Hall, Gainesville, P.O. Box 117330, USA

**Keywords:** Social network, Social Support, Severe mental disorder, Multilevel analysis

## Abstract

**Background:**

For psychiatric service users suffering from severe mental disorders, the social support provided by personal social networks is essential for living a meaningful life within the community. However, the importance of the support received depend on the relations between the providers of social support. Yet this hasn’t been addressed in the literature so far for people with severe mental disorders. This article seeks to investigate how characteristics of service users with severe mental disorders, their social contacts, and the pattern of relationships between those contacts influence the distribution and provision of social support to people with severe mental disorders.

**Methods:**

We collected personal network data relating to 380 psychiatric service users from a random sample of health care providers in Belgium. We computed various measures of the structure of those networks and of the position of support persons within those networks. We conducted a multilevel analysis of the importance of the support provided by each support persons.

**Results:**

The results show that the more central a support person was in the network of a service user, the more important his or her support was considered to be by the service user. Also, the denser the network in which a support person was embedded, the less important was the support he or she offers, but only for hospitalised service users.

**Conclusion:**

These finding highlight the collective dimension of social support. We discuss the implications for the organisation of mental health care.

## Background

For users of psychiatric services who have severe mental disorders, social support is key to living a meaningful life within their community [1]. It provides access to emotional, material, and cognitive support, including help with managing diseases and limiting the consequences of stressful situations [2–5]. It helps to reduce symptoms [6, 7], avoid multiple inpatient admissions [8], and improve access to health care service providers [9]. Psychiatric service users, like the general population, access social support through their personal social networks. A person’s personal social network (or ego network) is the web of his or her social relationships, i.e. the people he or she is in contact with and the contacts between those people [10, 11]. The personal social network can have a direct or indirect impact on health or well-being, including through the provision of social support [12, 13]. Personal social networks have often been used in social support studies to operationalise social support [10]. For users of psychiatric services, the personal social network includes both relatives (informal support) and health or social professionals (formal support) who also offer important support in the perspective of living in the community [9]. Moreover, the collaboration between all the supporting persons (formal or informal) also determines the delivery of support [14].

The size, composition (e.g. relatives and professionals), and structure of a person’s social network are likely to determine the support a person receives. In the general population, a large social network is associated with increased access to social support [15, 16] and a diversified network offers more support of all types [17, 18]. Individuals who are embedded in a cohesive network, in which a large number of the contacts know each other, seem to receive more support [15, 18, 19], although other studies, on more specific populations (migrants), found no association between network cohesion and social support [20].

We know less about the influence of the personal social network on social support for psychiatric service users. For instance, one study showed that relationships with life partners, friends, and peers are more supportive than those with family and professionals [23] . The presence in the social network of people who are aware of the person’s mental health problems is predictive of a broader array of perceived support functions [23]. Another study, which looked at network density (the ratio of actual to possible relationships) and social support, found no association between them [24]. Research on access to social support for psychiatric service users has tended to focus mainly on the association between support and service utilisation or symptomatology, rather than on the actual provision of support [8, 21, 25–27]. Moreover, previous studies have mainly investigated the effect of the whole network characteristics on the support provided. For service users, however, social support is the product of individual and collective factors, and the interactions between them: the provision of support does not depend only on the characteristics of the network of the person receiving support (the “ego” in personal network terminology) or on the characteristics of the social contact providing support (the “alter”). It also depends on how an alter is embedded in the personal network of the ego he or she is supporting. In other words, the support that a specific alter provides to an ego is not independent of the support that other alters provide to that ego. When an alter is connected to many other alters, he or she is more likely to provide support than if he or she is disconnected from other alters [28]. For example, a study of the general population found that the more ties an alter has to the other alters in an ego’s network, the more likely the alter and ego are to provide support to one another in relation to labour and housing issues [19]. Another way to illustrate this interdependence of alters has to do with the composition of the alters in a network: alters in a network composed mainly of other similar alters are more likely to be supportive than those connected to dissimilar alters [18, 19, 29].

Few studies, however, have actually examined the interdependence of the social support provided to psychiatric service users by alters. Social support has mostly been analysed from the perspective of the ego, without taking into account the different roles played by different alters in supporting the ego. The structure of a network is likely to influence the provision of support by each alter in at least three different ways. Having many ties with the ego’s other alters (degree centrality) provides a particular alter with more and better information, the capacity to respond more effectively in the event of a crisis, and, potentially, a better understanding of the ego’s needs. Moreover, for an alter, strategic contact with alters from the ego’s different social circles (e.g. family, friends, work, or leisure) who do not have other contacts with each other (betweenness centrality) can also increase the support offered to the ego by providing faster access to information and making it possible to coordinate the actions of the other alters. We might, therefore, expect more central alters to be more supportive than less central alters (hypothesis H1).

Furthermore, being connected to other alters who are similar in terms of alter role (relatives and friends, mental health professionals, or other professionals) increases an alter’s level of investment in the ego and in the other alters, because similar alters have shared interests and needs [30, 31]. We can, therefore, expect alters with more relationships with other similar alters to offer more support (hypotheses H2).

The connectivity within an ego’s network is also likely to have an impact on the support offered by the alters. Network cohesion (or distributed connectivity), i.e. the presence or extent of ties among alters, may increases the level of pressure of the group on alters to provide support. Network centralisation (or centralised connectivity), i.e. the extent to which it contains a small number of central alters, may also improve the coordination of the delivery of support. Thus, an alter embedded in a cohesive network is likely to offer more support because of the influence of other alters and an alter embedded in a centralised network is likely to offer more support as the support it offers is coordinated with the other alters (hypothesis H3).

Taking these arguments from the existing literature into consideration, we aim to explore how the pattern of relationships between alters is associated with the support they provide to an ego (a psychiatric service user). We ask three main questions about the determinants of the social support provided to psychiatric service users:

(RQ1) Are alters in more central network positions more supportive?

(RQ2) Are alters with more relationships with other similar alters more supportive?

(RQ3) Are alters embedded in more cohesive networks or in more centralised networks more likely to offer support?

Those questions are important from a health services and community health perspective. They may help to explain why the level of support offered to service users with severe mental disorders by certain types of alters may differ depending on the structure of the service users’ personal networks. Furthermore, in the context of community care, studies like this one can help design interventions to support specific alters in their efforts to help service users with severe mental disorders to live meaningful and integrated lives within the community.

It should also be mentioned that the data collected within the framework of this study is rare given the cost of this type of collection. We recruited people suffering from severe mental health problems in a variety of life contexts, using a random sample (at the level of service providers and service users). We collected the structure of their personal social networks and the importance of the support provided by each member of their networks, from the users’ point of view. This is a unique form of data collection.

## Methods

### Survey design

**Fig. 1:**
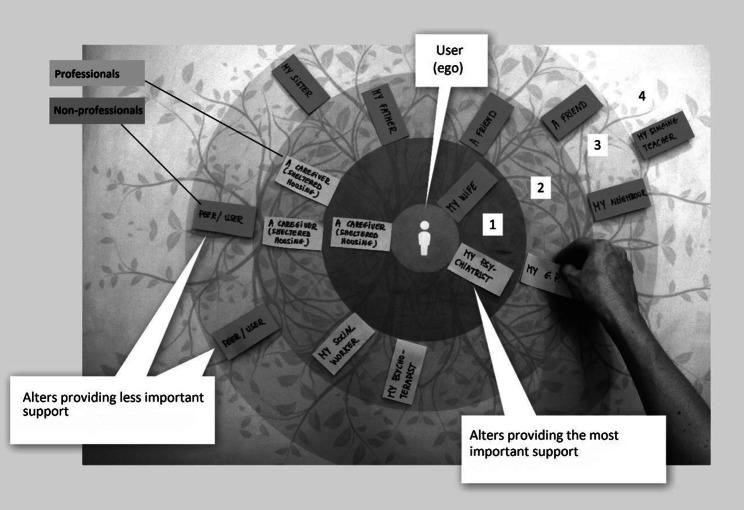
Collecting tool of users’ networks and the importance of the support provided by alters. (Morpheus Study, Belgium 2014–2015)

Inspired by recent developments in ego-network surveys in the domain of human services [32, 33], we developed a cross-sectional ego-network survey. An ego network consists of a single actor (ego, the respondent) and the individuals that ego is connected to (alters, the respondent’s social contacts) and all the links between those alters [10, 11]. The respondents were psychiatric service users and data were collected with the participant-aided sociogram technique developed by Hogan and colleagues [11, 34, 35] and a two-stage name generator [36]. First, we asked, “who are the people who support you?“ Then we explored the support in four specific domains of support: [1] administration and finance (“Who can support you in managing your money (invoices, budget, etc.) and in administrative matters (filling out documents, etc.)?“), [2] management of accommodation (“Who can support you in the management of domestic tasks or in moving to a new home, or take care of your things if necessary?“), [3] activities and relationships (“Who can support you in meeting people and finding activities or something to do during the day?“), and [4] health care (“Who can support you in managing your health and mental health (medication, hospitalisations, crisis situations, anxiety, etc.)?“). Once these two stages were complete, each respondent then placed alters on a bullseye map, using repositionable adhesive paper (Fig.1). The bullseye map contained four concentric circles, with the respondent represented at the centre. The respondent was asked to place alters (support providers) closer to or further from the centre according to the importance that he or she attached to the support offered by each alter. The instruction was: “Place people closer to you or further from you according to the importance you attribute to their support”. Alters in the first circle provided essential support, whereas alters placed in the last circle offered the least important support. The respondent was then asked to draw lines between alters on the bullseye map to indicate which alters exchanged information with each other about him or her (“who exchanges information about you?”).

### Respondents and sampling

We conducted this survey with a stratified clustered sample of 380 users of psychiatric services with severe mental disorders [37, 38] in three Belgian districts: one metropolitan area (east of Brussels), one post-industrial, underprivileged area (La Louvière-Manage), and one area with a largely service economy (Namur). In each area, we recruited in- and outpatient mental health service providers. The service providers were drawn at random from the directory of all available mental health and primary care service providers in the area of interest. A representative of the provider was asked to select service users with severe mental disorders from three strata: users living in a facility with 24-hour supervision, users living in collective housing, and users living in regular housing. The interviews took place in residential or outpatient care and, in a minority of cases, at home. The full survey and sampling design has been presented elsewhere [39].

### Measures

The dependent variable, the importance of the support received as perceived by the ego, was an ordinal measure corresponding to the concentric circle in which the alter was placed: most important (first circle, coded as 1) to least important support, as perceived by the ego (fourth circle, coded as 4) (See Fig.1). The participant-aided sociogram technique also uses concentric circles of this kind to collect similar information about the relationship between the ego and the alter (closeness) [34]. The independent variables were operationalised with two groups of variables at the alter level and at the network level. Variables at the alter level are used to answer the first two questions (RQ1 and RQ2), while variables at the network level are used to answer RQ3. Alter centrality was measured using degree centrality and betweenness centrality. Degree centrality is the number of ties to other alters, which shows the extent to which an alter knows other social contacts in the ego’s personal network, and is thus potentially able to access more information about the ego from other areas of his or her network. Betweenness centrality measures the number of times an alter falls on the shortest path between two other alters. Alters with high betweenness centrality are connected to more of ego’s different social circles and are more able to control the flow of information around the ego.

Also at the alter level, we calculated the percentage of alter-alter ties that each alter has within the group to which he or she belongs. We considered three groups of alters, corresponding to different roles or functions in a service user’s personal network: relatives and friends, mental health professionals, and other professionals (including general health care professionals, social service providers, the justice system, and generic non-health service providers). If that percentage exceeds 50%, the alter concerned has more relationships with people within his or her group than with out-group alters.

At the network level, we computed three indices of the connectivity between the alters, allowing us to describe the cohesion and centralisation of the network. Density, i.e. the number of ties existing between alters divided by the total number of possible ties, is a common measure of network cohesion in the literature on psychiatric service users’ networks [9, 14, 26, 40, 41]. Fragmentation, i.e. the proportion of alters that are not connected to each other directly or indirectly, is the opposite of density, but it also takes into account the indirect connections between alters (connections through other alters). Fragmentation determines how much opportunity there is for network members to communicate with each other, including through other network members [11]. Degree centralisation, finally, is the difference in degree centrality (number of alter-alter contacts) between the most central alter and the other alters, divided by the largest sum of differences that can exist in a network of the same size [42]. In other words, a network is considered to be more or less centralised depending on whether it contains more or fewer central actors.

We also calculated various indices that were useful as control variables. At the network level, we computed three indices: the network size, the proportion of professionals in the support network, and the number of different types of service providers involved. Other variables were collected at the ego level: age, psychiatric history (number of years since first contact with an outpatient or inpatient psychiatric service provider), and psychosocial functioning, which was collected by the Health of the Nation Outcomes Scale (HoNOS) [43, 44]. The HoNOS includes 12 items that address the severity of several psychosocial problems. All data were collected from service users, with the exception of the HoNOS, which was provided by a professional. Each user designated a professional who knew him or her well to fill in the scale [39].

### Statistical analysis

We first tabulated the different characteristics of the service users’ networks in relation to the importance of the support, using an F-test to assess the statistical significance of differences between support intensity levels. We then conducted a multilevel analysis of the importance of the support provided by each alter. We used cumulative logit models (or proportional odds ordinal logistic model) to predict the odds of a higher support level as opposed to a lower support level. Those considered the most supportive were placed in the support circle coded 1 and the least supportive were placed in the support circle coded 4. Thus, our models predicted the odds of an alter being in support circle 1 as opposed to 2, 3, or 4; in support circles 1 or 2 as opposed to support circles 3 or 4; etc.

**Fig. 2:**
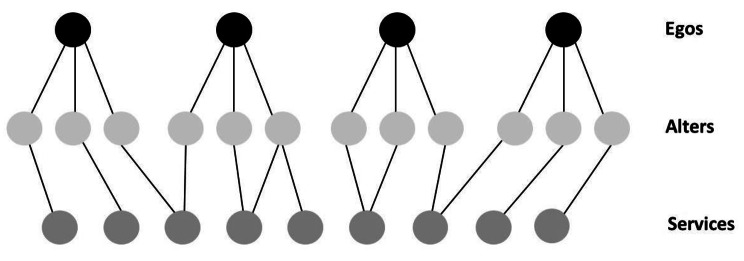
Structure of the non-hierarchical multilevel model

We used multilevel models, which are typical in ego-network analysis. Ego networks have a multilevel structure, as alters are not independent of each other and some egos could be clustered [45–47], as in this study, in which some users were recruited in similar services. This study has a cross-classified multilevel data structure: the relationship between ego and alter is clustered within two types of context at a higher level, the ego network on the one hand and the service provider to which alters belong on the other hand. Unlike most studies of personal networks, which have used hierarchical models with only one level of clustering [45, 48], we have two levels to take into account (ego network and alter’s service provider) and alters may belong to the networks of several egos, which creates a non-hierarchical multilevel structure (48) (Fig.2). To take into account the level of clustering of the alters within the ego network, we introduced a unique ego identifier into the model as a random intercept. Then, to take into account the level of clustering of the service providers to which each alter is linked, we associated each alter with a unique identifier for its service provider. Of the 2849 professional alters, only 226 (8%) could not be associated with a service provider. We associated each of these 226 with a specific individual service provider identifier. As most of these alters (56%) were independent practitioners (psychiatrists, psychologists, and general practitioners consulting privately, or lawyers, public guardians, and probation officers), this was an adequate solution. The other alters were human service providers unrelated to psychiatric care (housekeeper, social taxi, sports club, library, etc.). For relatives, we assigned a unique identifier to each extended family, considering that, like the professionals in a service provider, they are more likely to know each other and to exchange information about the user. We attached a unique identifier to each friend, as we cannot assume that they know each other. This unique “service provider” identifier was also added to the model as a random intercept, taking into account the fact that each alter is clustered within a service provider in addition to being clustered within an ego’s network (see Fig.2).

We also had to consider the possibility that an alter may be included in the networks of multiple egos. Indeed, it is often the case that the same care provider supports several service users. Among the user networks we collected, this was frequently the case for service users who used the same service providers. The model had to take that situation into account. We therefore sought to identify the alters that were nominated by multiple egos, in order to give each alter a unique identifier, independently of the egos with which it was associated. We thus reduced the 4602 alter nominations to 3808 unique alters: the same 327 alters were named 1199 times. It was not possible, however, to identify with certainty the identity of the alters associated with 448 nominations (9.7% of the 4602 nominations). Thus, for the purposes of the analysis, these 448 nominations were also considered as unique alters. We introduced this unique alter identifier in our non-hierarchical model as a random intercept.

*,*

On that basis, we built four models. The first includes the individual characteristics of the alters, i.e. their centrality (degree and betweenness), their alter role (mental health professionals, other professionals, relatives, and friends), and the percentage of their relationships that are within their own group. The second model includes the characteristics of the ego network, i.e. density, degree centralisation, and fragmentation. The third and fourth include the control variables. The third includes the network size, the proportion of professionals in the support network, and the number of different types of service providers involved; the fourth includes the ego’s characteristics (age, social functioning, and psychiatric history). The last model, the fifth, includes all the significant characteristics of the three previous models.

In building our non-hierarchical multilevel model, it became apparent that it was not possible to introduce into our model both the unique identifier of the alters and the unique identifier of the service providers: as the alters are nested within the service providers, it is difficult to separate their influence on the model from that of the service providers. We therefore introduced the two variables independently, in order to compare their respective influences. Table1 shows a reduction in deviance from 9882.38 to 9777.53 to the benefit of the model that included the service provider identifier as a random effect. Consequently, we used the service provider identifier as a random intercept in the following analyses. If some alters were cited by several egos, that was often related to the fact that the egos were supported by the same service provider. The introduction of the service provider identifier in the model is therefore likely to take duplicate citations of alters into account. In summary, we used a cross-classified model in which the units of analysis were the alters and those units were cross-classified into service providers (service provider identifier random intercept) and ego networks (ego identifier random intercept).

**Table 1:** Difference between a model including the identifier of alter and of each service provider

	Model I		Model II	
	**Coeff.**	**s.e.**	**Coeff.**	**s.e.**
**Variance components**				
Ego	0.70014	0.08960	0.7698	0.1056
Alter	0.07135	0.07158		
Service provider			0.8221	0.1342
-2logLikelihood	9882.38		9777.53	

*Morpheus Study, Belgium 2014–2015 (n = 380)*

Many of the indices used, however, whether at the alter or network level, are likely to be correlated with hospitalisation status. For example, a hospitalised service user is in contact with a larger number of professionals as a consequence of hospitalisation and those professionals are likely to know each other, which makes the network denser. Professionals in the hospital are also more likely to be considered as supportive, as hospitalised people are, at that time, vulnerable and in need of support. We therefore also stratified the previous analysis according to the respondents’ hospitalisation status at the time of the interview. We divided the service users we met into two groups. The first, composed of 179 users, contained those who were interviewed within hospital psychiatric services and the second, composed of 201 users, was made up of those who were met outside hospital and were not hospitalised at the time. We then estimated the same complete multilevel model for each of the two groups.

Calculations of the measurements for social networks were carried out in R, using Igraph, and the statistical analyses were carried out using SAS.

## Results

The users we met were, on average, 45.4 years old (std = 11.6) and had, on average, 12 years of psychiatric history (see Table2). They had a moderate score of 12.8/48 on the HoNOS scale (proxy for psychosocial functioning). At the time of the interview, 46.98% of them were hospitalised. The average user had a small network (mean = 12, std = 5.2 ) with low density (only 22% of all pairs of alters were directly connected to each other) and a high degree of fragmentation (65% of all pairs of alters were not connected to any other alters, directly or indirectly).

**Table 2:** Clinical features and characteristics of social support networks of psychiatric service users

	Mean or %	std	Min.	Max.
**Service user characteristics**				
Age (years)	45.4	11.7	19.0	78.0
Social functioning (HoNOS)	12.8	7.3	0.0	36.0
Psychiatric history (years)	11.7	11.0	0.2	64.0
Hospitalised at the time of the interview (%)	47.0			
**Size and composition of service user’s network**				
Network size (no.)	12.1	5.2	2.0	42.0
Professionals (%)	64.6	17.4	18.2	100.0
Types of services providers (no.)	3.9	1.9	1.0	11.0
**Structure of service user’s network**				
Network density (%)	22.2	17.9	0.0	100.0
Network degree centralisation (%)	22.6	12.0	0.0	66.7
Network fragmentation (%)	65.3	27.0	0.0	100.0

*Morpheus Study, Belgium 2014–2015: mean (or %) and std (n = 380)*

Most alters were located in the categories providing the most important levels of social support, as perceived by the service user (levels 1 or 2); a few were mentioned in the least important category (4.4% in level 4 of social support) (see Table3). Alters were either providers of mental health services (43.7%) or relatives and friends (38.1%); they had an average of 2.6 ties with other alters. Alters were, on average, 1.2 times in a broker position between two other alters and were mostly related to the same type of alters (63.9%).

Providers of mental health services and family and friends were most often assigned to level 1; those working in general health service providers, social service providers, the justice system, and generic non-health service providers had a more uniform distribution across the levels of importance of support as perceived by service users. The centrality of alters was higher for alters providing more important social support than for those providing less important social support. In the highest level of support (level 1), alters had on average 3.09 contacts; this decreased to 1.13 for the lowest level of support (level 4). The percentage of relations with similar alters was higher (68.4%) for the alters perceived as providing the most important support and it was lowest for the alters perceived as providing the least important support (41.7%). The analysis also shows that level 1 alters, who offered the most important support to the ego, were more often found in denser and less fragmented networks, with more professionals but fewer different types of service providers, than alters offering less important support.

The results of the multilevel multinomial regression are provided in Table4. Models I to IV model social support per block of covariates and Model V includes all covariates. From the first model, at the level of alter characteristics, we can note that the more central an alter was (in terms of degree or betweenness), the greater the importance given to that alter’s support by the ego. The percentage of relationships that an alter has with other members of his or her group yielded no significant results. Regarding the characteristics of the network (Model II), only the density of relationships in an alter’s network has a positive influence on the importance of the support offered by that alter to ego. Regarding control variables (Models III and IV), it appears that an alter in a network composed of a greater number of professionals or a smaller number of types of services providers was likely to offer more important support to ego. Moreover, being a relative or friend also put an alter in a better position to support ego. In contrast, none of the ego characteristics (age, social functioning, psychiatric history) seem to be associated with the importance of the support offered by an alter to the ego. The fifth model includes all covariates. It is the centrality of the alters, above all, that influences the importance of the support offered. The more central an alter was, the more important was the support it offers to the ego. In this model, the structure of the network in which an alter is involved seems to have no influence on the importance of his or her support. Regarding the composition of the network, the higher the percentage of professionals in an alter’s network and the fewer different types of services providers, the greater the importance of the support that alter offered to the ego.

In the analysis stratified by type of service user, degree centrality had a greater impact on the importance of the support provided to non-hospitalised service users than on that provided to hospitalised service users (see Table5). Whether an alter was a relative or friend was slightly less important when the service user was not hospitalised. The percentage of professionals in the service users’ networks was positively associated with the importance of the support provided to hospitalised service users, but that association was not significant for non-hospitalised service users. The presence of fewer service providers in the user’s network was associated with more important support from network members, whether the user is in or out of hospital, but that association is more important and more significant for those in hospital. Moreover, the density of relationships within the network was negatively associated, albeit not significantly, with the importance of the support offered by network members. The denser the network in which an alter was embedded, the less important was the support he or she offers.

**Table 5:** Multilevel analysis of structure of social networks according to perceived support, by hospitalisation status

	All service users, network and alter characteristics, by hospitalisation status at the time of the interview						
	**Hospitalised (n = 179)**				**Out of hospital (n = 201)**		
**Covariate**	**Coeff.**	**p value**	**s.e.**		**Coeff.**	**p value**	**s.e.**
**Alter characteristics**							
Alter degree (no.)	0.087	***	0.018		0.195	***	0.031
Alter betweenness (no.)	0.028	*	0.011		0.032		0.019
Mental health professionals (ref. categ.: other professionals)	0.176		0.283		0.076		0.17
Relatives and friends (ref. categ.: other professionals)	1.149	***	0.276		0.656	***	0.162
**Structure of service user’s network**							
Network density (%)	-0,892	*	0.428		-0.0008		0.536
**Composition of service user’s network**							
Professionals (%)	2.235	***	0.366		0.606		0.359
Types of service providers (no.)	-0.104	***	0.026		-0.093	**	0.029
**Random intercept**							
Ego	0.938	***	0.201		0.906	***	0.218
Service provider	0.602	***	0.211		0.95	***	0.238

*p < 0.05 **P < 0.01

***P < 0.001

Morpheus Study. Belgium 2014–2015 (n = 380).

## Discussion

This study investigated the importance of the support provided by alters to service users with severe mental disorders. The main results of these analyses are as follows: first, the more central an alter was in the ego-network of a service user, the more important his or her support was considered to be by the service user. Secondly, being in contact with other similar alters had no impact on the importance of the support delivered by an alter. Finally, the denser the network in which an alter was embedded, the less important was the support he or she offers, but only for hospitalised service users.

Our first main result allows us to answer our first question (“Are alters in more central network positions more supportive?”) in the affirmative. This supports the idea that a central position within a user’s network gives a comparative advantage to the alter, allowing him or her to offer more important support to the user, perhaps thanks to quicker and more complete access to information, a more comprehensive view of the user’s situation, a better understanding of the ego’s needs, and potentially faster and more adequate interventions. Central alters are also in a structural position that allows them to coordinate the support interventions of other alters. This is consistent with the literature, which shows that when an alter is connected to many other alters, he or she is more likely to provide support [28, 49]. While there is no literature specifically related to mental health that has addressed this issue, the role of the central alter is recognised within the widespread practice of case management [50–54]. Case management coordinates services providers, distributes responsibilities to the different alters, and facilitates information-sharing [55]. Also regarding the structural position of an alter within the ego network, our second main result leads us to answer our second question (“Are alters with more relationships with other similar alters more supportive?”) in the negative. Having more connections with people in the same category has no impact. What seems to be important in order for an alter to provide support is that they have a lot of contacts, not that they are in contact with similar alters. On one hand, similar alters connected to each other may share similar perspectives and may have stronger ties because of the common features, leading to more effective social support. Yet, on the other hand, strongly connected similar alters may provide redundant information and sub-optimal information, thus decreasing the added value of each alter. This has been discussed in previous experimental studies related to the adoption of behaviours [56]. Although being connected to similar others is frequent in social support network and is a key driver in the network formation, it may not increase the social support importance as perceived by ego.

Our third question is about the influence of the general structure of a user’s network on the importance of the support offered by the alters (and not the specific position of those alters in the network): “Are alters embedded in more cohesive networks or in more centralised networks more likely to offer support?”. The overall structure of the network does not affect the importance of the support provided by each alter. For hospitalised persons, however, the importance of support decreased as the density of relationships within the network increased. At first glance, this result appears to contradict another of our results, which shows that, for hospitalised persons, a higher percentage of professionals in a users’ network is associated with more important support from the members of that network. Indeed, having more professionals in an inpatient context is likely to lead to a denser network, as those professionals generally work in teams. This could mean that, in a very cohesive network where professionals coordinate with each other in formal and informal ways, the importance of the support is more equally distributed among alters, so that no single alter is perceived by the ego to be very important.

There are no studies similar to ours, which studies the importance of the support provided by alters (as perceived by ego) in relation to the structural position of alters in the networks of psychiatric service users, to which we could refer. Some studies, however, which look more generally at the structure of the networks of psychiatric service users, are helpful in interpreting these results [9, 14, 26, 40, 41, 57]. Cohesive networks have the potential to mobilise more supportive resources, partly because such networks make it easier for the alters to become aware of the importance of the problem [40] and also make it possible for them to compare their impressions with those of others [14]. There is evidence of this outside the field of mental health: individuals who are embedded in dense networks receive more support in both routine and crisis situations [18, 28, 58–63]. A dense network, however, could have a negative influence on the support offered by the members of a user’s network. High density may favour the dilution of responsibility, leading alters to believe that others would be equally able to offer support [14]. A dense network could also have consequences for the perception of the user who, rather than being supported, may feel constrained. A dense network limits interpersonal distance and ensures that information about a user’s situation is shared with almost everyone in the network, without the user being able to control possible distortions of the information that circulates about him or her [14]. Therefore, whereas during hospitalisation a large proportion of professionals within a network makes everyone feel more supported by improving the standard of support within the network, a very dense network, essentially organised around hospital resources, is likely to reduce the user’s perception of the importance of the support and even cause a rupture with the service provider [14].

### Limitations

This study has significant limitations. First, the networks were described by the users themselves, because there is no centralised information system in Belgium which allows us to describe the social support networks of users in an objective way. Users may not be aware of all the relationships between his or her support persons. However, as users have to identify relationships between people they know and have identified as supports persons, they are likely to be able to report them [10]. In addition, as the users we met may suffer from delusions and hallucinations, it is possible that the data we collected were not always totally accurate. This should, however, have a limited impact on our findings. Of the psychiatric service users we met, several expressed delusions, but only one person mentioned a person from his delusions in the list of support persons. The very process of data collection, based on very concrete questions, rooted in everyday life, seems to keep delusions at bay. At the same time, users may not remember all the support persons and may be unaware of certain exchanges between others, or may assume the existence of relationships between some of them. Elsewhere [39], Kappa coefficients indicated quite a good level of agreement between users’ reports and main clinicians’ reports of users’ social support networks. Other studies have shown that the methods of collecting ego-centric networks used here are reliable. Indeed, it seems that the information reported by ego about his or her alters is generally accurate [64]. Adams & Moody were able to show that there was 80% agreement between different egos about the same alter-alter social ties [65]. A recent study among elderly people with cognitive impairment found moderate to high levels of agreement between focal participants and study partners, who were nominated by each participant, in terms of their perceptions of focal participants’ networks [66].

Secondly, the cross-sectional design is vulnerable to reversed causation and unobserved confounders. The health professional who recommended users for participation in the study is likely to be influential. Their relationship may be a determining factor in the user’s willingness to participate and may also influence his or her responses, since the data also relate to the service provider that brought the user into contact with the researchers. Furthermore, our study focuses on the structure of relationships between the members of users’ networks. We did not focus on the content of the social interactions that occur within users’ networks. The same network structure can have quite different effects depending on whether the person’s social environment legitimises the way he or she assumes his or her social roles. Information on the content of the exchanges (shared values, for example) or the context in which they appear could also help us interpret our results. In “self-affirming social environments” [62], for example, a support person’s perception of the service user’s situation is likely to increase the impact of that person’s centrality on the support offered to the service user.

## Conclusion

These results can be useful for decision-makers, service users, and professionals alike. From an organisational point of view, these results point to the need to acknowledge and support people who are in a central position within the networks of psychiatric service users. They suggest that the development of individual or team case management practices should be encouraged. They also suggest that support should be provided to family carers, who are often under-recognised for their informal coordination work. In this respect, further research is needed to determine whether central actors offer more or less support depending on some of their characteristics, in particular whether they are professional or not. Concerning the clinical relationship between caregivers and service users, it would be useful if professionals could describe the networks of the users they support using the measurements used here and act accordingly. This would enable them to identify those people considered to be the most supportive and those with whom it would be useful to coordinate. From the user’s point of view, such a process would allow users to put forward their point of view and become involved in the organisation of their care. Furthermore, the ambiguous results associated with the density of relationships within the networks of hospitalised service users suggest that professionals should be particularly attentive to the potential effects of communication between alters on ego.

**Table 3:** Description of alters in the ego’s social support network, by perceived support

		Importance of support					
	**Total**	**Perceived support, level 1 (highest)**	**Perceived support, level 2**	**Perceived support, level 3**	**Perceived support, level 4 (lowest)**	**F Value / Khi-2**	
Number of alters: % (no.)	100 (4602)	50.7 (2326)	31.9 (1464)	13.0 (596)	4.4 (200)		
**Alter characteristics**							
Alters from mental health service providers: % (no.)	43.7 (2013)	50.7	34.0	12.1	3.2	182.1	***
Alters from general health service providers: % (no.)	7.8 (359)	35.0	39.5	17.6	7.8		
Alters from social service providers and justice system: % (no.)	8.2 (379)	38.5	30.2	21.5	9.8		
Relatives and friends: % (no.)	38.1 (1754)	57.9	27.9	10.3	3.8		
Alters from generic non-health service providers: % (no.)	2.1(97)	26.8	39.2	30.9	3.1		
**Alter structural position in ego network**							
Alter degree: mean no. (std)	2.6 (3.2)	3.1	2.1	1.6	1.1	52.5	***
Alter betweenness: mean no. (std)	1.1 (5.2)	1.5	1.0	0.5	0.4	8.0	***
Relationship within same group: mean % (std)	63.9 (46.0)	68.4	65.2	51.2	41.7	39.4	***
**Characteristics of the network in which alter is embedded**							
Network size: mean no. (std)	14.3 (6.3)^(1)^	14.3	14.5	14.7	13.2	3.1	*
Professionals: mean % (std)	61.9 (16.7)^(1)^	62.5	62.1	60.3	58.3	6.0	***
Types of services providers: mean no. (std)	4.2 (2.1)^(1)^	4.0	4.3	4.5	4.2	12.0	***
Network density: mean % (std)	20.9 (16.2)^(1)^	22.3	20.5	17.6	16.4	19.7	***
Network degree centralisation: mean % (std)	22.6 (11.2)^(1)^	22.8	22.6	22.2	21.8	0.7	
Network fragmentation: mean % (std)	66.3 (25.3)^(1)^	65.0	66.1	69.5	72.6	9.3	***

[1] These averages have as denominator the number of alters and not the number of egos. They are different from the averages presented in Table1

*p < 0.05 **P < 0.01

***P < 0.001

Morpheus Study, Belgium 2014–2015 (N = 4602)

**Table 4:** Multilevel analysis of structure of social networks according to perceived support

		Model I-IV^1^						Model V						
		**Alter, network (size and composition, structure), and service user characteristics**						**All characteristics**						
**Covariate**		**Coeff.**		**p value**		**s.e.**		**Coeff.**		**p value**		**s.e.**		
**I. Alter characteristics**														
Alter degree		0.169		***		0.023		0.19		***		0.022		
Alter betweenness		0.045		***		0.011		0.044		***		0.011		
Relationships within the same group (%)		0.208				0.118								
Mental health professionals (ref. categ.: other professionals)		0.058				0.181		0.067				0.177		
Relatives and friends (ref. categ.: other professionals)		0.9		***		0.158		0.96		***		0.156		
**II. Structure of service user’s network**														
Network density (%)		2.632		**		0.696		-0.622				0.534		
Network centralisation (%)		-0.19				0.674								
Network fragmentation (%)		0.657				0.48								
**III. Size and composition of service user’s network**														
Network size (no.)		0.005				0.013								
Professionals (%)		1.169		**		0.410		1.615		**		0.438		
Types of service providers (no.)		-0.144		***		0.036		-0.124		**		0.04		
**IV. Service user characteristics**														
Age (years)		0.006				0.005								
Social functioning (HoNOS)		0.011				0.008								
Psychiatric history (years)		-0.005				0.006								
**Random intercept**														
Ego		0.688		***		0.102		0.958		***		0.146		
Service provider		0.855		***		0.147		0.652		***		0.145		

^1^ This column presents four blocks of variables introduced separately in the model

The random intercept mentioned in this column is related to the fourth block.

* p < 0.05 **P < 0.01.

***P < 0.001.

Morpheus Study, Belgium 2014–2015 (n = 380).

## Data Availability

The datasets used and/or analysed during the current study are available from the corresponding author on reasonable request.
